# 2-Deoxy-D-Glucose: A Novel Pharmacological Agent for Killing Hypoxic Tumor Cells, Oxygen Dependence-Lowering in Covid-19, and Other Pharmacological Activities

**DOI:** 10.1155/2023/9993386

**Published:** 2023-03-02

**Authors:** Raman Singh, Vidushi Gupta, Antresh Kumar, Kuldeep Singh

**Affiliations:** ^1^Division Chemistry & Toxicology, WTL-Clean and Renewable Energy Pvt. Ltd., New Delhi, India; ^2^Department of Chemistry, Indian Institute of Science Education and Research, Mohali, Punjab, India; ^3^Department of Biochemistry, Central University of Haryana, Jant-Pali, Mahendergarh, Haryana 123031, India; ^4^Department of Applied Chemistry, Amity University Madhya Pradesh, Gwalior, MP 474005, India

## Abstract

The nonmetabolizable glucose analog 2-deoxy-D-glucose (2-DG) has shown promising pharmacological activities, including inhibition of cancerous cell growth and N-glycosylation. It has been used as a glycolysis inhibitor and as a potential energy restriction mimetic agent, inhibiting pathogen-associated molecular patterns. Radioisotope derivatives of 2-DG have applications as tracers. Recently, 2-DG has been used as an anti-COVID-19 drug to lower the need for supplemental oxygen. In the present review, various pharmaceutical properties of 2-DG are discussed.

## 1. Introduction

**Figure 1:**
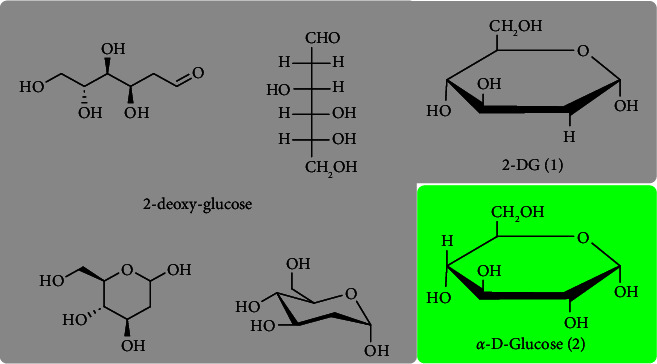
Structure of 2-deoxy-D-glucose (CAS: 154-17-6), synonyms: 2-DG; 2-deoxy-D-arabino-hexose; D-arabino-2-deoxyhexose.

2-Deoxy-D-glucose (2-DG, 2-deoxy-D-arabino-hexopyranose) is a natural [1], nonmetabolizable glucose analog and a competitive inhibitor of glycolysis [2] in which the 2-hydroxyl group is replaced by hydrogen (Figure 1). 2-DG blocks the activity of different enzymes involved in glycolysis, leading to cell death. Hyperglycemic condition aggravates cancer cell proliferation, inflammatory conditions, and viral infection [3]. In this review, the pharmaceutical properties of 2-DG have been discussed. This review article describes the pharmacological properties specific to 2-DG and its isotopic derivatives but excludes substituted derivatives of 2-DG.

### 1.1. Drug-likeness of 2-DG

2-DG has a molecular weight of 164.158 Da, logP of −1.525, five hydrogen bond acceptors (HBA), and four hydrogen bond donors (HBD). Thus, there are four matching Lipinski's rules. The polar surface area (PSA) of 2-DG was 90.15, and it consisted of one rotatable bond (RotB). Thus, two matching Veber's rules exist.

### 1.2. Toxicity of 2-DG

Toxic effects of 2-DG result from its ability to block glycosylation [4, 5] but not glycolysis [6]. Ketogenic Diet increases tolerance against glycolysis inhibitors [7]. Experimental results show that 2-DG is a relatively harmless compound at low doses, but that it can lower blood pressure and slow breathing at higher doses [8]. Most of the studies indicate that a clinically tolerable dose of 2-DG is up to 63 mg/kg/day [9]. Beyond this several side effects are observed including reversible hyperglycemia, gastrointestinal bleeding, and QTc prolongation [9]. However, recent studies carried out by DRDO use a higher dose of 90 mg/kg/day [10]. Exposure to 2-DG causes cytotoxicity and radiosensitization via a mechanism involving changes in thiol metabolism and these effects may be more prominent in transformed vs. normal cells [11].

## 2. Analytical Methods for Detection and Determination of 2-Deoxy-D-Glucose

The concentration and purity can be measured in a sample of crystalline or liquid material by HPLC with accuracy and precision suitable for the analysis of active pharmaceutical ingredients and drug products [12]. The method is suitable for the standardization and quality control of APIs and drugs [12]. UV-HPLC (195 nm) has been used to detect and quantify 2-DG using a *μ*Bondapak 10 *μ*m NH_2_ column and a Varian MicroPak 10 *μ*m NH_2_ column. The retention time is usually four minutes with an eluent 85% MeCN/H_2_O [13]. Polymer-based amino column (HILICpak VG-50 4E column) and Shodex SUGAR SC1011 columns have also been used to separate 2-DG and glucose. Pharmacokinetic studies of 2-DG involve the estimation of 2-deoxyglucose in the plasma [14]. For this purpose, precolumn fluorescent derivatization was achieved by reductive amination of 2-DG using sodium cyanoborohydride and 2-aminobenzoic acid [14].

## 3. Pharmaceutical Profile of 2-DG

**Figure 2:**
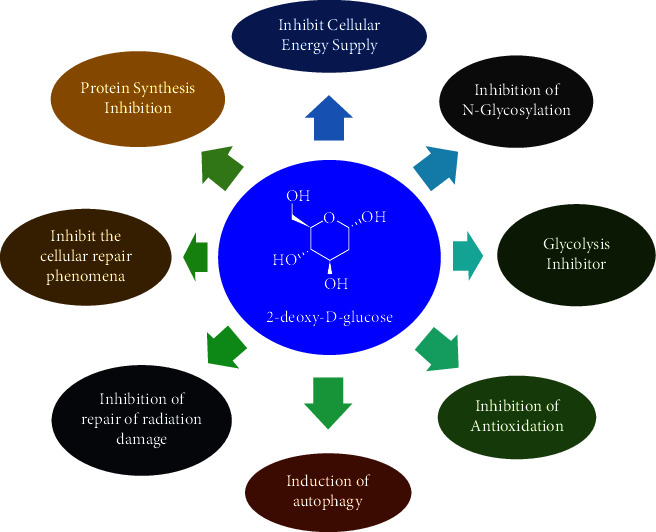
Pharmacological activities of 2-DG.

**Figure 3:**
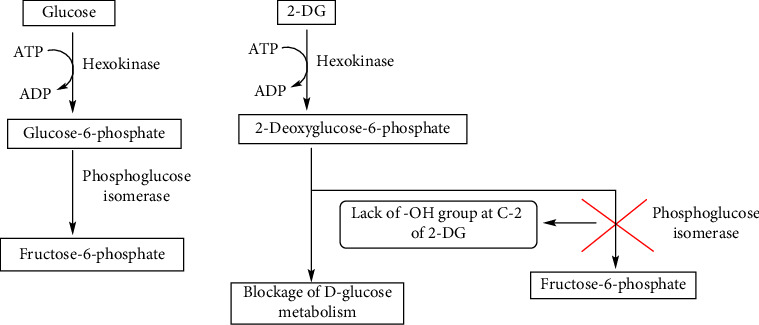
Mechanism of action of 2-DG.

The molecule 2-DG follows Lipinski's rule of five and has several activities, kills hypoxic tumor cells, and lowers oxygen dependency in case of Covid-19 (Figure 2). A number of studies have described different biological activities [3], but it is not approved as a drug until May 2021 [15]. In May 2021, 2-DG was found an emergency use as an anti-Covid-19 drug allowing patients to recuperate more quickly by lowering the need for supplemental oxygen. 2-Deoxyglucose (2-DG) is a toxic glucose analog. 2-DG has a pleiotropic mechanism of action (Figure 3) [16–18].

### 3.1. Inhibition of N-Linked Glycosylation Process

**Figure 4:**
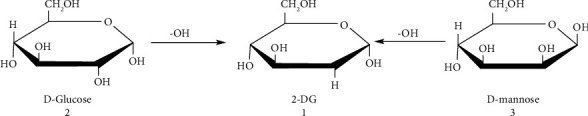
Preparation of 2-DG from D-glucose and D-mannose.

2-DG also contains a structural resemblance to mannose, which strongly interferes in the N-linked glycosylation process, which resulted in to halt in protein synthesis and causes ER stress. As D-glucose and D-mannose are epimers at C-2, the deoxygenation at C-2 gives one identical product, i.e., 2-DG (Figure 4). Therefore, 2-DG affects D-mannose metabolism, including glycosylation processes [16], and induces endoplasmic reticulum stress [16, 19]. 2-DG stimulates autophagy, enhances oxidative stress, and suppresses N-linked glycosylation [20].

2-DG mimics mannose has brought up the prospect of developing it as an antiviral agent, in addition to restricting cancer growth [21]. Glycolytic inhibitors such as 2-DG potentiate the activity of Paclitaxel [22].

### 3.2. Inhibition of Inflammation

Recent reports suggest the usefulness of 2-deoxy-D-glucose in the inhibition of inflammation [23], controlling respiratory infections, and treatment of human genital herpes [24]. 2-DG shows anti-inflammatory activities through the regulation of anti-inflammatory mediators and the polarization of macrophages [3]. During ocular infection with herpes simplex virus (HSV), stromal keratitis occurs due to inflammatory reactions in the eye. *In vivo* experiments to limit glucose utilization using 2-DG showed diminished lesions with fewer proinflammatory effectors [25]. 2-DG also acts as a potential energy restriction mimetic agent (ERMA) [26], and has application in reducing acute inflammation events caused by pathogen-associated molecular patterns (PAMPs) [26], 2-DG application as ERMA in drinking water can help to avoid pathogenic exposure-induced inflammatory events, which can help to prevent both acute and chronic inflammatory illnesses [26]. In the mice model, treatment with 2-DG (0.4% w/v in drinking water) reduces infiltration of inflammatory cells, inflammatory signaling activation, oxidative stress, capillary damage in lungs [26], and reduced the BALF and serum [26]. 2-DG reduces the inflammatory responses triggered by glycogen accumulation caused by coal dust because macrophages have reconstituted glycogen metabolism [27]. A study on dextran sulfate sodium-induced colitis-mouse (mouse model for inflammatory bowel disease), alleviated laminarin-induced arthritis in the SKG mouse (model for human rheumatoid arthritis), LPS shock (model for a cytokine storm), and LPS-induced pulmonary inflammation (model for COVID-19) suggested that 2-DG is effective for the treatment of various inflammatory disease due to its capability to inhibit cytokine receptor glycosylation [28].

### 3.3. Hypoxic Suppressor of Cancer Cell Growth

It is well documented that cancer cells utilize more energy than normal cells, and they favor glycolysis for ATP production and cell metabolism. Utilization of glycolytic energy by tumor cells even in the presence of oxygen is called aerobic glycolysis or the Warburg effect. This glucose metabolism in tumor cells showed a promising target for the regulation of the growth of cancerous cells. 2-DG competes with the binding of glucose to hexokinase (HK), which converts into 2-DG-6-phosphate (2-DG-6-P) by phosphorylation. The accumulation of 2-DG-6-P in cells inhibits phosphoglucose isomerase (PGI) activity. Thus, 2-DG limits glucose uptake and the downstream metabolic pathway, which depletes the ATP level and induces cell death.

Several applications of 2-DG are known for the efficient elimination of cancer cells, and the synergetic effect of 2-DG has been reported in the literature [29]. The limited therapeutic effect of 2-DG in cancer treatment is overcome by its beneficial synergistic anticancer effect with other therapeutic agents or radiotherapy [20] by blocking glycolysis in hypoxic tumor cells and subsequent cell death [30]. 2-DG manipulates its similarity to glucose and the tendency of cancer cells to utilize glycolysis even in the presence of oxygen, a process known as aerobic glycolysis or the Warburg effect [31, 32]. 2-DG acts as a D-glucose mimic, suppressing glycolysis by forming and accumulating 2-deoxy-D-glucose-6-phosphate (2-DG6P) inside cells, blocking hexokinase and glucose-6-phosphate isomerase, and causing cell death (Figure 2) [33].

Nanoparticles or nanosized molecules show the pharmaceutical effect differently. Nanoliposomes have been used in various drug delivery systems. 2-DG-containing nanoliposomes have shown inhibition of glycolysis in cancer cells. The synergistic effect of these 2-DG-loaded liposomes with the coloaded drug enhances mitochondrial depolarization and subsequent apoptosis [34].

Studies have shown that 2-Fluoro-Deoxyglucose resembles glucose more closely than 2-DG. Lampidis employed a QSAR-like method combined with a flexible coupling strategy to determine that the analog binding affinities to hexokinase I decrease as the size of the halogen increases [30].

2-fluoro-2-deoxy-D-glucose (2-FG) >2-chloro-2-deoxy-D-glucose (2-CG) > 2-bromo-2-deoxy-D-glucose (2-BG).

D-glucose had the highest affinity binding affinity to hexokinase I, followed by 2-FG and 2-DG [30]. 2-DG dramatically increased ATP depletion and 4E-BP1 phosphorylation. On the other hand, high amounts of 2-FDG can prevent further protein glycosylation processes by competing with glucose [35].

When the energy supplied to the cell decreases, 2-DG causes metabolic stress, which inhibits immune cells' (leucocytes) actions and causes downregulation of immune-relevant genes (CSF-1R, NCCRP-1, Hep, TCR-a, IgMH, MHC-II, C3, and IL-1). [36] On DU145 cells and mouse xenograft tumors, 2-DG combined with buforin IIb causes greater inhibition of proliferation, higher arrest of the G1 cell cycle, and more apoptosis than either treatment alone [37].

Several drugs have been tested in combination with 2-DG to inhibit the growth of cancer cells. These drugs and corresponding cancer types are listed in Table 1.

### 3.4. Autosomal Dominant Polycystic Kidney Disease (ADPKD)

Autosomal dominant polycystic kidney disease (ADPKD) is characterized by defective glucose metabolism. Chiaravalli studied the effect of low doses of 2-DG on ADPKD progression in orthologous and slowly progressive murine models created postnatally by inducible inactivation of the Pkd1 gene. These studies established proof-of-principle support for the use of 2-DG as a therapeutic strategy in ADPKD [72]. 2-DG can suppress the activity of seizures and retards the epilepsy progression *in vitro* as well as *in vivo* [73].

The combination of metformin and a low dose of 2-deoxyglucose synergically inhibits cyst formation and human polycystic kidney cell proliferation [74, 75]. Cheong and his colleagues reported that 2-DG with metformin can prevent tumor growth in mouse xenograft models [76].

Due to its similarity in the structure of glucose and nonparticipation in glycolysis [77], 2-DG has emerged as a tool for metabolism-independent GS investigations [78, 79]. Studies on the effect of 2-DG treatment on the mHypoE-29/1 cell line suggest that the metabolism-dependent GS pathway is responsible for glucose detection in neuronal cells and the downregulation of AgRP mRNA levels.

The effect of exposure to 2-DG on AgRP mRNA levels in the adult mHypoA-NPY/GFP model and the embryonic model showed that both cell lines expressed Tas1R2 and Tas1R3 mRNA transcripts, indicating the involvement of metabolism-independent glucose sensing mechanisms. These studies also suggest that control of AgRP mRNA expression in embryonic cells is metabolism-dependent, whereas adult cells may act in a metabolism-independent manner [80].

2-DG is an antagonist of glucose metabolism and targets hypoxic cancer cells resistant to chemotherapy by induction of apoptosis [81], 2-DG inhibits angiogenesis [82], and 2-DG inhibits metabolism [21].

2-DG inhibits T-cell-mediated cytolysis since 2-DG metabolites compete with glucose metabolites for key enzymes (such as glycosyltransferases) that are necessary for cytolysis expression [83].

The synthesis and characterization of CyNE 2-DG, a new NIR fluorescent DG analog, was reported by Vendrell et al. The coupling of 2-deoxy-glucosamine and tricarbocyanine carboxylic acid in the presence of the coupling reagent, i.e., HATU results in the formation of CyNE 2-DG [84].

Immobilization and 2-DG-induced central neuroglycopenia should be identified as different types of stressful stimuli, causing their effects through different neural pathways, based on secretory, hemodynamic, and synthesis of adrenal catecholamine rate responses [85].

The suppression of proteoglycan production by the GAG chain could be linked to ATP depletion in cells. In confluent primate VSMCs, the ATP content of cells decreased by 25–30% after exposure to 2-deoxyglucose. ATP levels and proteoglycan synthesis recovered to baseline after 2-DG was removed [86]. 2-DG inhibits substance P (SP) production in the bodies of sensory ganglion cells of vagal cells, as well as its bidirectional transit to the CNS, thoracic, and abdominal viscera [87]. Total protein synthesis was not affected when the ratio of hexose to 2-DG was 20 1 or greater. Under similar conditions, invertase and acid phosphatase production and secretion are inhibited by 2-DG. Glucan formation was also inhibited. The mechanism of inhibition of total uptake of external sugar by 2-DG after a lag period involves intracellular 2-DG-6-P, which directly inhibits the conversion of fructose-6-P to glucose-6-P and mannose-6-P by phosphohexose isomerases; simultaneously decreasing the transport of fructose or maltose into cells [88–90].

2-DG inhibits cellular repair phenomena, even after completing the unscheduled DNA synthesis [91–94]. 2-DG inhibits DNA repair and the repair of potentially lethal damage in cancerous cells. These two phenomena were studied in respiratory-deficient yeast cells irradiated with *X* assayed by unscheduled DNA synthesis and cell viability after irradiation, respectively [92–94].

LPS-induced aerobic glycolysis is inhibited by 2-DG; therefore, collagen synthesis is also inhibited [95]. Glycolysis suppression results in decreased muscle protein synthesis as a result of decreased basal mTORC1 signaling [96].

### 3.5. Antiseizure Effects and Retarding Effect in Epilepsy Progression

2-DG exhibits antiseizure effects through the netrin-G1-KATP signaling pathway by upregulating K (ATP) subunits kir6.1 and kir6.2 [97, 98]. In animal models, it retards the progression of epilepsy [99]. The epileptic brain shows dynamic metabolic changes. Focal zones of onset of seizures are hypometabolic during the interictal period and hypermetabolic during seizures [100]. Therefore, glycolysis plays an important role in these dynamic metabolic changes; therefore, 2-DG could abrogate seizure activity and retard epilepsy progression.

### 3.6. COVID-19 and 2-DG

Perris hypothesized the use of modified sugars for the treatment of viral infections in 2007 [101]. Later it was found that 2-DG inhibits the propagation of epidemic diarrhea virus [102] in human rhinoviruses [103], pandemic SARS-CoV-2 [103, 104], and endemic human coronaviruses [103]. These *in vitro* studies suggested broad-spectrum antiviral use of 2-DG [103]. 2-DG limits viral proliferation in the body by selectively killing cells infected with the SARS-CoV-2 virus by halting energy production and viral synthesis [104]. 2-DG has limited use in SARS-CoV-2 patients suffering from stroke, hypoxic-ischemic encephalopathy, and other critical illnesses [105–107]. In Phase II clinical trials, the addition of 90 mg/kg/day of 2-DG to the standard of care (SOC) for the treatment of moderate to severe COVID-19 demonstrated clinical benefit over SOC alone [107].

The Drugs Controller General of India (DCGI) has approved the emergency use of 2-deoxy-D-glucose (2-DG, 1) on 1 May 2021 [15]. 2-DG is used as an adjunct therapy in patients with mild to serious COVID-19 to recover quickly by reducing the supplemental oxygen requirement.

#### 3.6.1. Docking and Computational Studies

COVID-19 disease has emerged as an epidemic of the 21st century due to the nonavailability of effective antiviral agents, as well as its pathophysiology [108]. The fragment molecular orbital (FMO) method has been used to characterize SARS-CoV-2 S-protein binding interactions with the ACE2 and B38 Fab antibodies involved in ACE2-inhibitory binding. These studies helped to understand the amino acid residues critical for molecular recognition between the S-protein and the ACE2 or B38 Fab antibody [109]. The binding of 2-DG and 1,3,4,6-tetra-O-acetyl-2-deoxy-D-glucose to viral main protease 3CLpro and NSP15 endoribonuclease is studied using molecular coupling techniques. These studies show that viral receptors are inactivated due to the formation of a hydrogen bond between 2-DG and proline residues. Furthermore, 1,3,4,6-Tetra-O-acetyl-2-deoxy-D-glucose forms a hydrogen bond with the glutamine amino acid residues of the viral spike glycoprotein [110]. The coronavirus disease 2019 (COVID-19) pandemic has highlighted the value of FDG-PET/CT in diagnosis [111, 112]. Molecular coupling and molecular dynamics simulations showed that 2-DG had a positive interaction with SARS-CoV NSP12 and the SARS-CoV-2 RBD spike-ACE2 complex [113].

Drug-drug interactions (DDI) and synergistic regulatory potential have been investigated in a report describing a molecular coupling and simultaneous molecular dynamics simulation of multiligands to study the combined effect of 2-DG with other 62 selected drugs and phytochemicals. In terms of binding energy, the combination of 2-DG with Ruxolitinib, Telmisartan, and Punicalagin was superior to that of the selected individual compound [114].

#### 3.6.2. Similarity between the Pathophysiology of Cancer Cells and SARS-CoV-2 Infected Cells with Respect to Glycolysis Inhibitors

Cancer cells and virus-infected cells have similarities; both require a large amount of energy because of their very high proliferation rate. This high energy requirement increases glucose uptake by infected cells and uses glycolysis and glycosylation for energy production [115]. It promotes mitochondrial signaling, i.e. aerobic glycolysis. Hyperglycemic conditions, such as diabetes, facilitate the invasion and propagation of SARS-CoV-2 and an aggravated immune response [116, 117]. Thus, impaired glucose metabolism will destroy infected cells and viruses in parallel with cancerous cells. Infected cells hungry for energy will absorb a high amount of glucose antimetabolite compared to normal cells. These facts became the basis of the use of 2-DG in the treatment of COVID-19 [106, 118].

#### 3.6.3. Upregulation of Glycolysis Results in Mortality in COVID-19

COVID-19 is a serious acute respiratory disease associated with cardiovascular complications. The interaction of virus Nsp6 with host proteins from the MGA/MAX complex (MGA, PCGF6, and TFDP1) was studied by expressing the SARS-CoV-2 protein in the hearts of *Drosophila* and using transcriptomic data. This interaction blocks the antagonistic MGA/MAX complex, which shifts the balance to MYC/MAX and activates glycolysis. Nsp6-mediated upregulation of glycolysis disrupts cardiac mitochondrial function, which increases ROS in heart failure. This could explain the cardiac pathology associated with COVID-19. Inhibition of glycolysis with 2-deoxy-D-glucose reduces the Nsp6-induced cardiac phenotype in flies and mice. These findings suggest glycolysis as a pharmacological target for COVID-19-related heart failure [119].

Thirumalaisamy reported Hyaluronic acid-2-DG conjugated as a novel drug in the treatment of COVID-19 [120]. Replication of SARS-CoV-2 in Caco-2 cells was prevented by inhibiting glycolysis and nontoxic 2-DG concentrations [121]. SARS-CoV-2 replication requires high energy and is supported in colon cancer cells by increased carbon metabolism. Thus, glycolysis inhibitor 2-DG inhibits SARS-CoV-2 replication [121].

### 3.7. Resistance to 2-DG

Overexpression of the odr1 gene of *Schizosaccharomyces pombe* produces a strong resistance to 2-DG with different resistance mechanisms for budding yeast and fission yeast [122]. *S. pombe* Odr1 hydrolase can act in the toxic form of 2-DG, similar to *Saccharomyces cerevisiae* Dog1/Dog2, which encodes HAD-like hydrolase enzymes and exhibits specific 2-DG-6- phosphatase activity [122].

### 3.8. Other Applications

The use of 2-DG with low-dose radiation therapy is also suggested for anticancer, anti-inflammatory, and antibacterial/antiviral effects [123]. 2-DG is also used as a hypoglycemic stimulus [66]. 2-DG has been used to assess glucose uptake activity by measuring the intracellular accumulation of 2-DG [124]. [^14^C]-deoxyglucose ([^14^C]-2-DG) is used as a glucose tracer. The method has been used to trace the exchange of glucose between plasma and brain and its phosphorylation during hexokinase glycolysis in tissues [125]. Direct evaluation of local cerebral metabolic activity is possible by using [^14^C]-2-DG in the quantitative autoradiographic method. This method provides access to quantitative measurement of glucose utilization and histological identification of affected cortical areas [126].

PET is used to measure glucose metabolism using the 2-DG method proposed by Sokoloff et al. Similarly to 2-DG, F18-labeled deoxyglucose is also an analog of glucose and is taken up in the brain and is phosphorylated in glycolysis. The absence of a C-2 hydroxyl prevents glycolysis; thus, it builds up intracellularly.

PET measures arterial tracer concentration, glucose uptake kinetics, and the regional cerebral glucose metabolic rate (rCMRGlc) can be measured or calculated. Positron computed tomography (PCT) and (F-18)2-fluoro-2-deoxy-D-glucose (FDG) are used to measure the local cerebral metabolic rate of glucose [127, 128]. The PCT method is used to measure glucose uptake in breast cancer cells [129]. The method is also used for the study of brain cells [130], lung cells [131], upper airway inflammation [132], infection in the case of multiple ventriculoperitoneal shunts [133], and myocarditis-STREAM [134].

18F-fluoro-2-deoxy-D-glucose has been used in positron emission tomography (FDG-PET) with computed tomography (CT) for lymph node (LN) staging in urothelial carcinoma (UC) [135], follicular lymphoma [136], polymyalgia rheumatica [137], thyroid nodules [138], and detection of bone marrow metastases [139, 140].

## 4. Synthetic Methods

The synthesis of 2-DG has been described and reviewed in the literature [141–144]. Selected recent methodologies have been discussed here. As D-glucose and D-mannose are epimers in C-2, deoxygenation in C-2 gives an identical product, i.e., 2-DG (Figure 4) [145].

**Figure 5:**
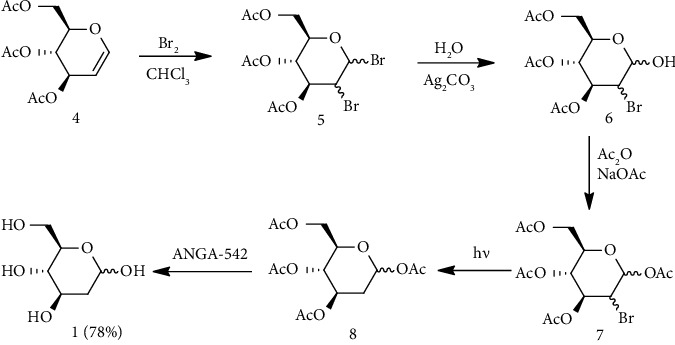
Preparation of 2-DG by photolysis of *α* and *β* anomers of **7**.

Glucal is the glycal formed from glucose and is one of the common starting materials for the synthesis of 2-DG. A general conversion involves the bromination (or halogenation) of Glycal at C-2 followed by the replacement of bromine with hydrogen. Bromination takes place in nucleophilic solvent using molecular bromine. Binkley et al. reported photolysis of *α* and *β* anomers of **7** to yield *α* and *β* anomers of **8**. Treatment of **8** with Baker ANGA-542 ion exchange resin in methanol produced 2-DG with a yield of 78% yield [141]. Compound **7** was synthesized by nucleophilic bromination of **4** followed by hydrolysis and acetylation (Figure 5) [141].

**Figure 6:**
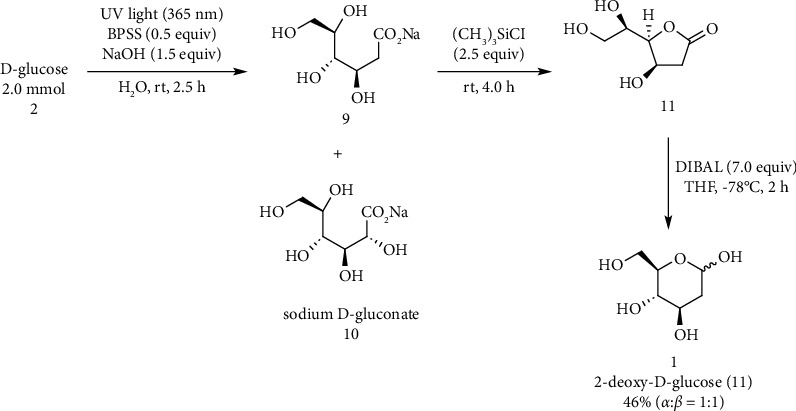
Synthesis of 2-deoxy-D-glucose (1).

Masuda and coworkers reported the synthesis of 2-DG from D-glucose. 2-Deoxy-D-glucose was prepared in three steps from natural D-glucose dispensing with any protection/deprotection procedure and was obtained in 48% yield (Figure 6) [146].

**Figure 7:**
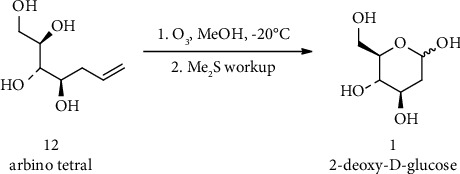
Synthesis of 2-DG by ozonolysis of tetrols **12**.

Roush and coworkers synthesized 2-DG by ozonolysis of tetrols **12**. Tetrols **12** was generated by methanolysis of the corresponding tetraacetates (Figure 7) [147].

**Figure 8:**
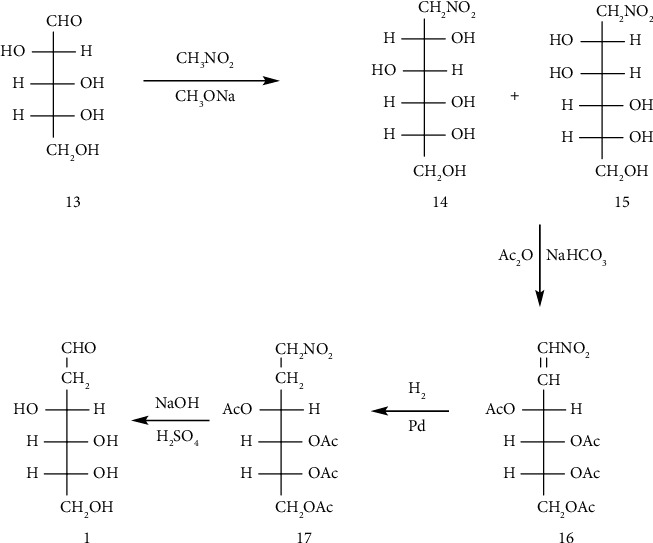
Synthesis of D-arabo-2-desoxyhexose from ribose.

Sowden and his coworkers reported the synthesis of D-arabo-2-desoxyhexose from ribose **13** (Figure 8) [148].

**Figure 9:**
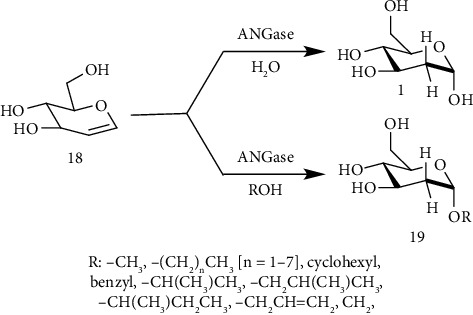
Synthesis of alkyl *α*-D-2-deoxyglucosides by using *Aspergillus niger α*-glucosidase.

2-DG was produced in 95% yield by using a multistep procedure using Rabbit muscle aldolase (RAMA, D-fructose-1,6-diphosphate aldolase). RAMA catalyzed the reaction of 1,3-dioxane-2-acetaldehyde with DHAP followed by dephosphorylation with AP producing a ketone. The NaHB(OAC)_3_ mediated reduction of the ketone gave a mixture of diastereomers in a 2 : 1 ratio in 75% yield. The 5S isomer was resolved to give acetal in 55% yield. The deprotection of the acetal with aqueous 1.0M HCI/THF (1 : 1) yielded 2-deoxy-D-arabino-hexose (95%) [149]. Kim and coworkers (Figure 9) synthesized alkyl *α*-D-2-deoxyglucosides (A2DGs) by using *Aspergillus niger α*-glucosidase (ANGase) [150].

## 5. Future Perspectives

2-DG has been investigated for combination therapy to inhibit cancerous cells. However, the recent use of 2-DG to treat COVID-19 patients under emergency conditions has opened up new hope for the development of new antiviral medicines. COVID-19 is a viral disease caused by SARS-CoV-2 variants. Mutation in the virus is of much concern, which results in the deactivation of available drugs and monoclonal vaccines. Thus, a target that is not directly affected via mutation has its own value. Targeting glycolysis in energy-hungry infected cells will stop the multiplication of the virus [117, 119, 151]. Further, all variants of SARS-CoV-2 follow rewired glycolysis, which makes 2-DG as a good starting point for the development of broad-spectrum antiviral [103]. Normal cells could rely on the citric acid cycle and oxidative phosphorylation using Acetyl-CoA from Fatty acids and ketone bodies. The application of 2-DG is dose-dependent, thus safe doses should be determined by their effects.

## 6. Conclusions

2-DG is a dual D-glucose and D-mannose mimetic and exploits increased glucose metabolism to kill glycolytic cells. The biological effects of the molecule 2-DG include inhibition of sugar uptake, inhibition of glucose metabolism, antiviral, anti-inflammatory, anticancer, antiepileptic activity, and more. It has found use as a tracer, cancer diagnostic tool, and metabolite inhibitor. Positron emission tomography has made use of 18F-fluoro-2-deoxy-D-glucose. Due to its ability to reduce inflammation and kill glycolytic cells, 2-DG exhibits activity against SARS-CoV-2. Antiviral properties of 2-DG have paved the way for the development of new antiviral drugs and therapies for hyperglycemic patients. Further investigation is necessary to determine the safest doses for various applications, the mechanism of action, the toxicity, and the interactions of 2-DG with various edibles.

## Figures and Tables

**Table 1:** Drugs used in combination with 2-DG to inhibit cancerous cells.

Cancer type	Combined therapy with 2-DG	Studies	References
B cell lymphoma cells	Metformin	Preclinical	[38–41]

Bladder cancer	Cisplatin	Preclinical	[42–44]
	Doxorubicin	Preclinical	[42, 45–47]
	Gemcitabine	Preclinical	[42]

Breast cancer	Metformin	Preclinical	[38–41]
	NCL-240	Preclinical	[48]
	Doxorubicin	Preclinical	[42, 45–47]
	Daunorubicin	Preclinical	[49]
	Fenofibrate (FF)	Preclinical	[50]
	Mito-Q, Mito-CP, Dec-TPP+	Preclinical	[51]
	Trastuzumab	Preclinical	[52]
	Radiotherapy	Preclinical	[11, 53, 54]
	Virotherapy (avian Newcastle disease virus (NDV))	Preclinical	[55]
	Docetaxel	Clinical	[9]

Cervical cancer	Radiotherapy	Preclinical	[11, 53, 54]

Cervical carcinoma	Alpha-tocopheryl succinate	Preclinical	[56]

Colon adenocarcinoma	Alpha-tocopheryl succinate	Preclinical	[56]

Colon cancer	Daunorubicin	Preclinical	[49]

Ehrlich ascites tumor-bearing mice	Etoposide	Preclinical	[57]

GBM	Cisplatin	Preclinical	[42–44]
	Metformin	Preclinical	[38–41]
	Oligomycin	Preclinical	[58, 59]
	Bevacizumab	Preclinical	[60]

Head and neck cancers	Docetaxel	Clinical	[9]

Head and neck carcinoma	Cisplatin	Preclinical	[42–44]

Hepatocellular carcinoma	Sorafenib	Preclinical	[45, 61, 62]

Leukemia	Barasertib and everolimus	Preclinical	[63]

Lung adenocarcinoma	Alpha-tocopheryl succinate	Preclinical	[56]

Lung cancer	Berberine	Preclinical	[64]
	Docetaxel	Clinical	[9]

Melanoma	NCL-240	Preclinical	[48]

Melanoma	Fenofibrate (FF)	Preclinical	[50]

Neuroblastoma	Resveratrol	Preclinical	[65]

Non-Hodgkin lymphoma	Methylprednisolone	Preclinical	[66]

Non-small cell lung cancer	Adriamycin	Preclinical	[67]
	Paclitaxel	Preclinical	[67]
	Afatinib	Preclinical	[68]

Non-small cell lung carcinoma	Ferulic acid with irradiation	Preclinical	[69]

Osteosarcoma	Adriamycin	Preclinical	[67]
	Paclitaxel	Preclinical	[67]
	Fenofibrate (FF)	Preclinical	[50]

Ovarian cancer	Metformin	Preclinical	[38–41]
	NCL-240	Preclinical	[48]

Pancreatic cancer	Salirasib	Preclinical	[70]
	50-Fluorouracil	Preclinical	[71]

Papillary thyroid carcinoma	Doxorubicin	Preclinical	[42, 45–47]
	Sorafenib	Preclinical	[45, 61, 62]

Prostate cancer	Radiotherapy	Preclinical	[11, 53, 54]

Small cell lung cancer	Oligomycin	Preclinical	[58, 59]

Small lung carcinomas	NCL-240	Preclinical	[48]

## Data Availability

Data sharing is not applicable to this article.
